# Alteration of Gut Microbiota After Antibiotic Exposure in Finishing Swine

**DOI:** 10.3389/fmicb.2021.596002

**Published:** 2021-02-12

**Authors:** Hee Eun Jo, Min-Sung Kwon, Tae Woong Whon, Doo Wan Kim, Misun Yun, Jieun Lee, Mi-Young Shin, Sung-Hak Kim, Hak-Jong Choi

**Affiliations:** ^1^Microbiology and Functionality Research Group, World Institute of Kimchi, Gwangju, South Korea; ^2^Department of Animal Science and Biotechnology, Chonnam National University, Gwangju, South Korea; ^3^Swine Division, National Institute of Animal Science, Rural Development Administration, Cheonan, South Korea; ^4^Department of Animal Science and Bioindustry, Chonnam National University, Gwangju, South Korea

**Keywords:** antimicrobial, fecal microbiome, swine, gut dysfunction, meta-analysis

## Abstract

Subclinical doses of antimicrobials are commonly used in the swine industry to control infectious diseases and growth performance. Accumulating evidence suggests that swine administered with antibiotics are susceptible to disease development due to disruption of the beneficial gut microbial community, which is associated with host immune regulation, nutrient digestion, and colonization resistance against pathogens. In this study, we found that finishing swine administered with lincomycin showed gut dysbiosis and increased diarrhea incidence compared with control swine. 16S rRNA amplicon sequencing was used to analyze the gut microbiota in finishing swine administered with lincomycin. The relative abundance of detrimental microbes, such as species of *Clostridium*, *Aerococcus*, *Escherichia-Shigella*, and *Corynebacterium* was increased in the feces of lincomycin-administered finishing swine, but that of bacteria associated with fiber degradation, such as species of *Treponema*, *Succinivibrio, Fibrobacter*, and *Cellulosilyticum* was decreased. Moreover, administration of lincomycin significantly increased the enrichment of metabolic pathways related to pathogenicity and deficiency of polysaccharide degradation. These results suggest that lincomycin treatment could cause severe disruption of the commensal microbiota in finishing swine.

## Introduction

Antibiotics are frequently used for growth promotion, disease prevention, or disease treatment in agriculture. The effects of antimicrobials on the improvement of growth rate and food efficiency were defined in the 1940s (Moore et al., 1946), and the addition of antibiotics to livestock feed has become a common practice. Given the production and maintenance costs, the use of antibiotics is very efficient, and experts predict that their use will increase in the future (Van Boeckel et al., 2015). Administration sub-therapeutic doses of antibiotics, such as ASP50 (Looft et al., 2012), Tylosin (Kim et al., 2012), and Carbadox (Looft et al., 2014) have been documented to have several advantages in swine health (Cromwell, 2002). However, indiscreet uses of antimicrobials cause the escalation of antibiotic-resistant bacteria and genes (Xiao et al., 2016; Gresse et al., 2017). Moreover, antibiotics adversely affect host health, such as antibiotic-associated diarrhea and indigestion (Young and Schmidt, 2004). *Clostridioides difficile* infection is the most common example of antibiotic-associated diarrhea in swine (Post and Songer, 2004).

Emerging evidence has shown that administration of antibiotics induces gut dysbiosis (imbalance of gut microbiota), and this imbalance results in the loss of colonization resistance (Stecher et al., 2013). Over 100 trillion microbes ecologically inhabit the mammalian intestinal tract, termed the gut microbiome, which contributing to the maintenance of metabolic functions in the digestive tract and interact with the host under physiological and immunological conditions (Macdonald and Monteleone, 2005; Nieuwdorp et al., 2014). Also, the commensal microbiota provides colonization resistance against pathogenic bacteria to maintain epithelial integrity (Stecher et al., 2013). The intestinal microbiota is modulated by several factors including host genetic factors, supplements, and age. Furthermore, oral antibiotic use is a critical factor that disrupts the gut microbiota balance (Gresse et al., 2017; Schmidt et al., 2018), and antibiotic-induced dysbiosis enables the expansion of pathogenic bacteria (Zeineldin et al., 2019), which infect healthy hosts. Despite this knowledge, administration of antibiotics is commonly practiced for efficient swine production. Therefore, a detailed understanding of the effects of in-feed antibiotics on the structure and function of the swine gut microbiome is required.

The late stage in swine production is the stage at which the animal reaches market weight, and infectious disease and poor growth performance during this period lead to mortality and delayed shipment. Lincomycin, the antibiotic used in this study, is a broad-spectrum antimicrobial that acts as an inhibitor of bacterial protein synthesis (Morar et al., 2009). This antibiotic is commonly used in the late stage of swine to treat and prevent infectious enteric diseases, such as ileitis and/or swine dysentery (Waack and Nicholson, 2018). As well as administration of subtherapeutic doses of lincomycin ameliorates growth performance in combination with other antimicrobials and supplements (Gong et al., 2008). However, recently, the possibility of adverse effects of lincomycin administration on swine health have been reported. High presence of lincomycin- and lincosamide-resistant genes has been observed in the gut of Chinese, French, and Danish pigs (Xiao et al., 2016), and the 100% incidence of diarrhea was observed in rats that were administered lincomycin by gastric gavage for 7 days (Lv et al., 2017). In addition, the dissemination of lincomycin-resistant genes were detected in surrounding environments adjacent to swine farms (Li et al., 2013).

Since limited information is available regarding the effect of lincomycin-induced changes on swine microbiome, we examined the differences in fecal microbial composition and predicted metabolic pathways between lincomycin-administered and non-administered finishing swine, by 16S rRNA gene sequencing. Furthermore, we combined our data with publicly available dataset of the fecal microbiome in swine prescribed other antibiotics, providing detailed information on the alterations in swine gut microbiome induced by diverse antimicrobial agents.

## Materials and Methods

### Sample Collection

**FIGURE 1:**
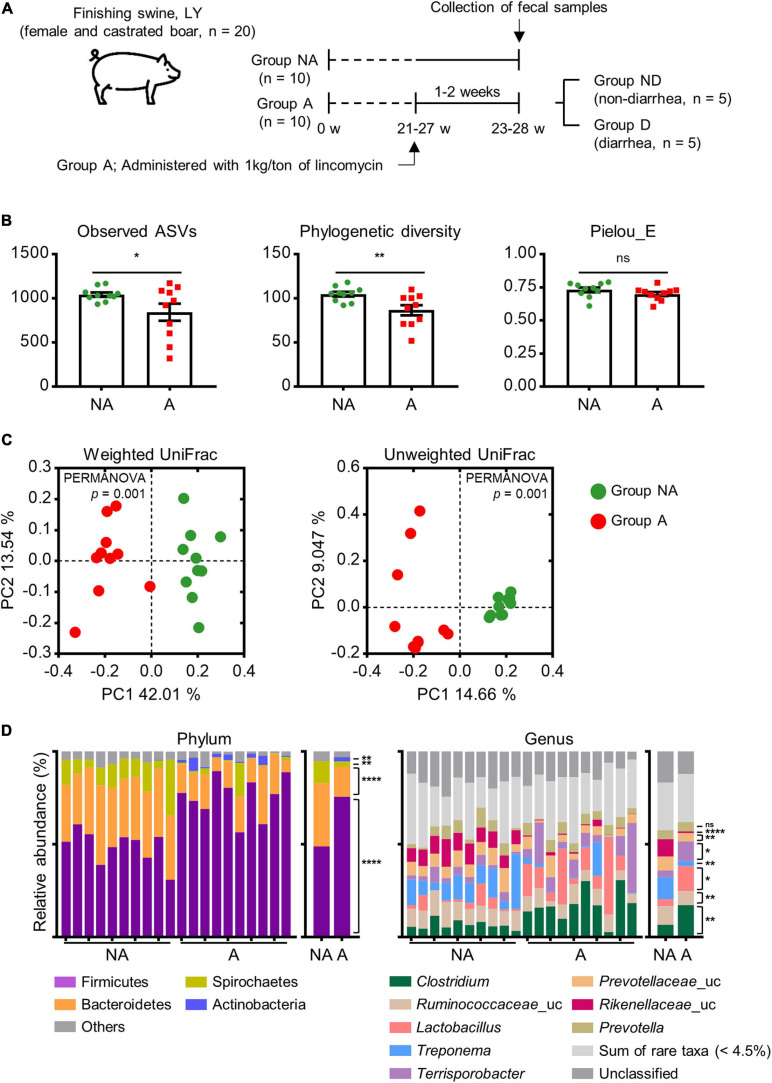
Experimental design and comparison of swine gut microbiota between groups administered (A) and not administered (NA) lincomycin. **(A)** All finishing swine were crossbred Landrace × Yorkshire (LY). Swine in the treatment group were administered a subclinical dose of lincomycin (0.1%, 1 kg/ton) through drinking water. NA, non-administered group; A, lincomycin-administered group. Samples from group A were further grouped depending on the occurrence of diarrhea: ND, no diarrhea; D, diarrhea. **(B)** α-diversity of swine fecal microbial communities between groups NA and A as analyzed by a one-tailed Student’s *t*-test (*p*-value * < 0.05, ** < 0.01). Values are expressed as mean ± SEM. Ns, non-significance. **(C)** β-diversity of swine fecal microbiota, calculated from PCoA plots based on the weighted and unweighted UniFrac distances. Statistical analysis was performed using PERMANOVA. **(D)** The relative abundance of the top 4 phyla and top 8 genera of fecal bacteria present in both groups. Abundance of significantly different bacterial phyla and genera were analyzed by a one-tailed Student’s *t*-test (*p*-value * < 0.05, ** < 0.01, **** < 0.0001). Uc, unclassified; ns, non-significance.

Twenty female and castrated male crossbred swine (Landrace × Yorkshire, 23–28 weeks of age) were fed a standard commercial corn-soybean diet. Ten finishing swine, which were not administered the antibiotic (hereafter referred to as group NA, *n* = 10), were raised at the National Institute of Animal Science (Cheonan, South Korea). The other ten finishing swine received the antibiotic (hereafter referred to as group A, *n* = 10) were raised on a local commercial farm (Muan, South Korea). They were treated with a subclinical dose of lincomycin (0.1%, 1 kg/ton) daily for 1–2 weeks through the feed. The animals were provided water and feed *ad libitum*. After 1–2 weeks treatment, fecal samples were immediately collected from each swine (Figure 1).

All fecal samples were immediately stored on ice after collection and transported to the laboratory, and then recorded according to the “Bristol stool form scale” to classify diarrhea cases (Lewis and Heaton, 1997). Statistical analysis between NA and A group for Bristol stool from scale was performed using a one-tailed Student’s *t*-test. The lincomycin administered swine were further grouped as non-diarrhea (ND, *n* = 5) and diarrhea (D, *n* = 5) groups following evaluation based on the Bristol stool form scale (Supplementary Table 1). The fecal samples were stored at −80°C until further use.

All experimental procedures were conducted by the guidelines approved by the Institutional Animal Care and Use Committee (IACUC) at the National Institute of Animal Science (approved no. NIAS-2019119).

### DNA Extraction From Feces and 16S rRNA Gene Amplicon Sequencing

Bacterial genomic DNA was extracted from the feces using the Fast DNA Spin Kit for Soil following the manufacturer’s protocol (MP Biomedicals GmbH, Heidelberg, Germany). DNA samples were quantified using a NanoDrop 2000 UV-Vis spectrophotometer (Thermo Fisher Scientific, Wilmington, DE, United States). Extracted DNA was stored at −80°C. Sample DNA was processed for 16S rRNA gene amplicon sequencing using a 250 bp paired-end protocol on the Illumina MiSeq sequencing system (Illumina, San Diego, CA, United States). Amplification was performed using the following barcoded primers targeting the V3–V4 region of the bacterial 16S rRNA gene. 314F, 5′-TCGTCGGCAGCGTCAGATGTGTATAAGAGA CAGCCT-ACGGGNGGCWGCAG-3′ and 805R, 5′-GTCTCGT GGGCTCGGAGATGTGTATAAGG-ACAGGACTACHVGGGT ATCTAATCC-3′. The MiSeq Reagent 500-cycle v2 kit (Illumina, San Diego, CA, United States) was used for sequencing 16S rRNA gene. 16S rRNA gene amplicon sequencing was carried out at ChunLab, Inc. (Seoul, South Korea).

### Sequence Processing and Bioinformatics Analysis

Illumina MiSeq demultiplexed FASTQ reads were imported into QIIME2 (version 2019.4^1^). The demultiplexed sequences were produced from chimeric sequences and singleton amplicon sequence variants (ASVs) were filtered with DADA2 (Callahan et al., 2016). Identified ASVs were aligned using MAFFT (Katoh et al., 2002) and further processed to construct a phylogeny with FastTree2 (Price et al., 2010). Alpha and beta diversity analyses were performed using the QIIME2 diversity plugin^2^. Alpha diversity was calculated with observed ASVs, phylogenetic diversity, and Pielou’s evenness indices. Principal coordinate analysis (PCoA) was performed with the q2-diversity plugin using weighted and unweighted UniFrac distance matrix. PCoA plots were created by GraphPad Prism v.7.05 software (GraphPad Inc., La Jolla, CA, United States) and statistical significance for the observed variations was assessed using the permutational multivariate analysis of variance (PERMANOVA) function with 999 permutations. Taxonomy was assigned to ASVs using the q2-feature-classifier (Bokulich et al., 2018), with a Naïve Bays classifier based on the Silva_132 99% (Quast et al., 2013), which has been trimmed to include the V3–V4 region of 16S rRNA gene, bound by the 314F-805R primer pair. This was applied to the paired-end sequence reads to generate taxonomy tables. Taxonomic and compositional analyses were performed using the feature-classifier plugins^3^, taxa^4^, and composition.

To identify discriminative taxonomic biomarkers, linear discriminant analysis (LDA) effect size (LEfSe) was performed with an LDA log score cut-off of 3.0, followed by the Kruskal-Wallis test with a Wilcoxon test cut-off of *p* < 0.05. An implementation of LEfSe, including a graphical interface incorporated in Galaxy framework is provided online at http://huttenhower.sph.harvard.edu/galaxy. This method for metagenomic biomarkers uses LDA to estimate the effect size (Segata et al., 2011).

PICRUSt2 (Phylogenetic Investigation of Communities by Reconstruction of Unobserved States) was performed to predict the functional pathways in KEGG Orthology (KOs) from the 16S rRNA gene sequencing data (Langille et al., 2013).

### Data Set Collection and 16S rRNA Gene Processing for Meta-Analysis

We performed a meta-analysis using publicly available 16S rRNA amplicon sequencing data from the NCBI. For inclusion in the meta-analysis, the studies should have used swine feces samples, Illumina MiSeq sequencing system, and the associated metadata. Two studies were included in the meta-analysis (SRP045387 and SRP115601). In SRP04538, the swine were fed chlortetracycline, sulfathiazole, and penicillin for 9 weeks, and in SRP115601, the swine were fed ampicillin, gentamycin, and metronidazole for 1 to 2 weeks. Detailed information of each study is provided in Supplementary Table 2.

All of the sequencing data files were identified through a literature search of the Short Read Archive (SRA^5^), downloaded using the SRA toolkit, and analyzed by QIIME2 (version 2019.4). We excluded samples with feature counts below 10,000 from the analysis (SRR5941318 and SRR5941314 in SRP115601 dataset) after DADA2 denoising. We then merged feature tables at the genus level by summing their respective abundances and calculated beta diversity metrics by using Bray-Curtis dissimilarity with q2-plugin. We used the non-phylogenetic and taxonomic annotation-based clustering methods (Bray-Curtis dissimilarity matrix), which we expected would minimize technical variations among studies (e.g., sequenced 16S rRNA gene variable region) as previously described (Kim et al., 2020). The PICRUSt2 analysis was performed in the same way as described above.

### Statistical Analysis

Each analysis was considered significance at *p* < 0.05 (one-tailed) and all data were presented as the mean ± standard error of mean (SEM) using GraphPad Prism v.7.05 software (GraphPad Inc., La Jolla, CA, United States). Alpha diversity and comparison of relative abundance were evaluated with a one-tailed Student’s *t*-test (*p* ^∗^ < 0.05 was considered statistically significant). Statistical differences between sample pairs or two groups of samples in KEGG pathway were analyzed using STAMP (Statistical Analysis of Metagenomic Profiles) software package (version 2.1.3) (Parks et al., 2014).

### Data Availability

The datasets generated for this study can be found in NCBI GenBank, accession numbers are PRJNA643361 (https://www.ncbi.nlm.nih.gov/bioproject/?term=prjna643361).

## Results

### 16S rRNA Gene Profiles in Swine With Lincomycin Administration

An average of 32,888 non-chimeric 16S rRNA gene sequences were available for analysis. All libraries had a saturated rarefaction curve and the sequences were denoised using DADA2 (Callahan et al., 2016). We assessed microbial alpha diversity based on the observed ASVs, phylogenetic diversity, and Pielou’s evenness diversity. We found that group A showed a significant decrease in richness (observed ASVs; *p* = 0.029, phylogenetic diversity; *p* = 0.005), but not in Pielou’s evenness compared with group NA (*p* = 0.09, Figure 1B). We next measured beta diversity using the weighted and unweighted UniFrac distance matrix. The results showed that the gut microbiota of group A were clustered separately from that of group NA (*p* = 0.001, Figure 1C), indicating that lincomycin administration altered fecal microbiota and diversity.

The ASVs mapped to 22 phyla, 39 classes, 84 orders, 186 families, and 475 genera. The top 4 phyla and top 8 genera are displayed in Figure 1D. Firmicutes and Bacteroidetes were the most prevalent phyla in both groups, followed by Spirochetes and Actinobacteria. These accounted for 95.08 and 96.87% of the reads in group NA and A, respectively. The abundance of Firmicutes and Actinobacteria was increased, while that of Bacteroidetes and Spirochetes was decreased in group A (*p* < 0.05). At the genus level, *Clostridium* and an unclassified *Ruminococcaceae* genus were the dominant genera in group A. Other major genera included *Lactobacillus*, *Treponema*, *Terrisporobacter*, an unclassified *Prevotellaceae* genus, an unclassified *Rikenellaceae* genus, and *Prevotella*; these genera accounted for more than 50% of total sequences. Sum of rare taxa represented that the sum of the genera with an average of less than 4.5% of relative abundance. The abundance of an unclassified *Rikenellaceae* genus and *Treponema* was decreased, while the abundance of *Terrisporobacter* and *Clostridium* was increased in group A (Figure 1D), indicating significantly differences at the genus level in gut microbial community structure of the two group.

### Significant Differences of Swine Fecal Microbiota After Lincomycin Administration

**FIGURE 2:**
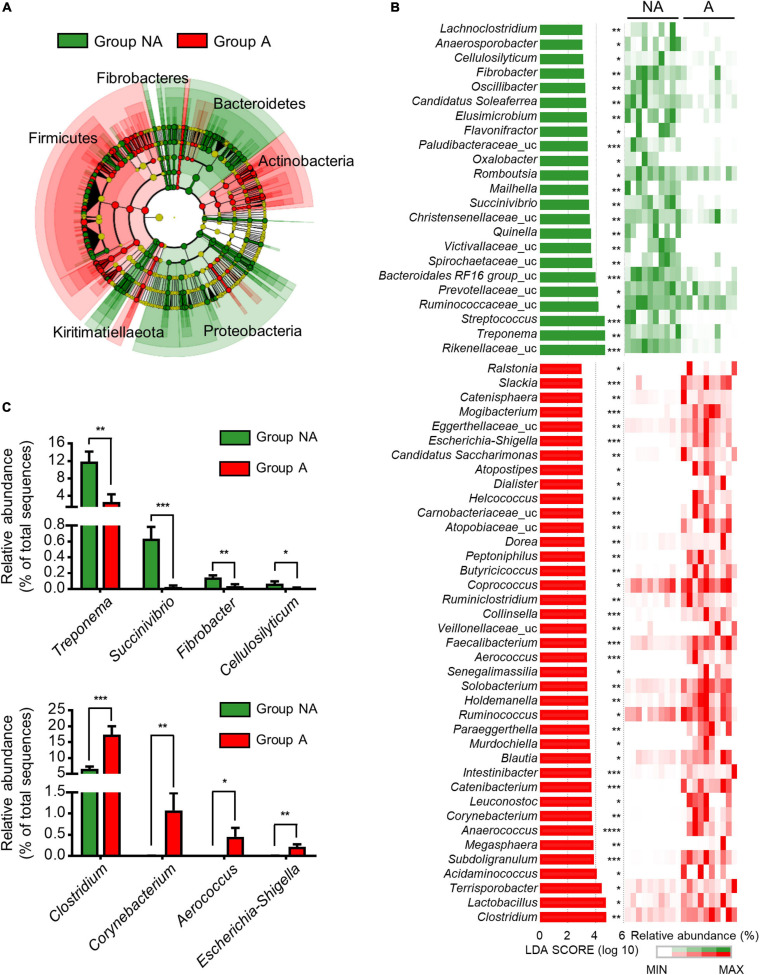
Comparison of swine microbial communities between the two groups. **(A)** Phylogenetic cladogram from LEfSe analysis, depicting the taxonomic association between the microbiome communities of groups NA and A. Each node represents a specific taxonomic type. **(B)** The ranking of significantly different genera by LEfSe method was revealed from the log LDA scores of the two groups. LEfSe was based on the non-parametric factorial Kruskal-Wallis sum-rank test followed by the Wilcoxon Signed-Rank test. Featured LDA scores >3.0 were plotted (*p*-value * < 0.05, ** < 0.01, *** < 0.001, **** < 0.0001). Uc: unclassified. **(C)** Bar plots illustrating selected features at the genus level. Values are expressed as mean ± SEM. Statistical analysis was performed using a one-tailed Student’s *t*-test (*p*-value * < 0.05, ** < 0.01, *** < 0.001).

LEfSe was used to further determine whether specific bacterial taxa were differentially enriched between the groups A and NA. A phylogenetic cladogram (Figure 2A) and histogram (Figure 2B) were generated. The cladogram presenting the taxonomic hierarchical structure of the fecal microbiota from phylum to genus indicated significant differences in phylogenetic distributions between the microbiota of groups NA and A (Figure 2A). Using a logarithmic LDA score cut-off of 3.0, we identified 23 and 39 genera that were relatively more abundant in the microbiota of group NA and A, respectively (Figure 2B). Several genera, including *Treponema* (*p* = 0.003), *Succinivibrio* (*p* = 0.0003), *Fibrobacter* (*p* = 0.003), and *Cellulosilyticum* (*p* = 0.05) were significantly over-represented in the feces of group NA, whereas *Clostridium* (*p* = 0.0005), *Corynebacterium* (*p* = 0.009), *Aerococcus* (*p* = 0.02), and *Escherichia-Shigella* (*p* = 0.003) were enriched in group A (Figure 2C), thus indicating remarkable difference in fecal microbiota between groups A and NA.

**FIGURE 3:**
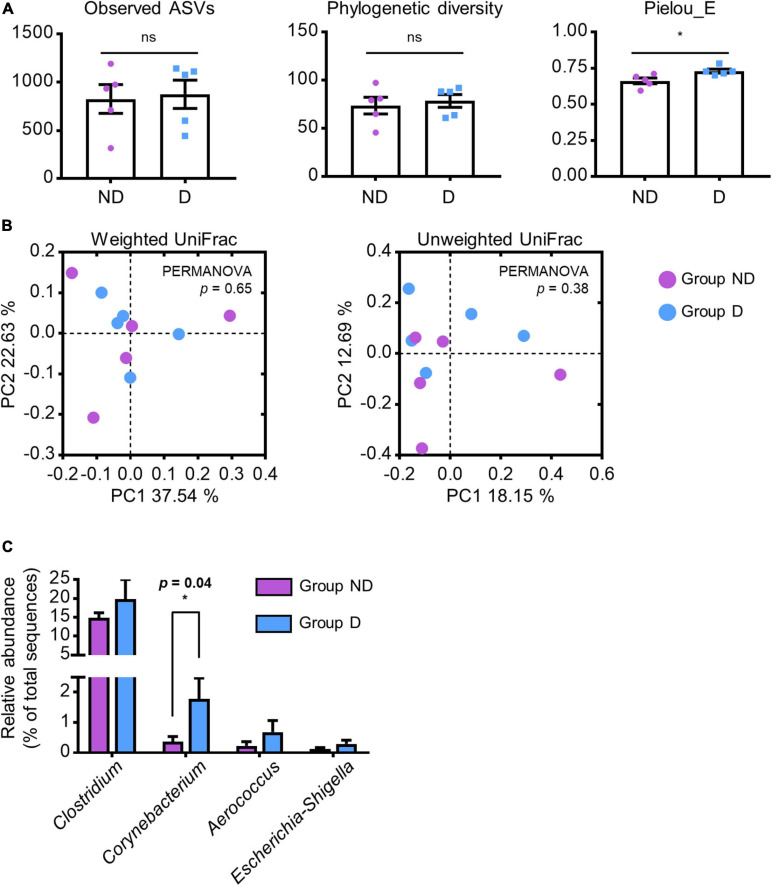
Alteration of specific antibiotic-susceptible microbes. **(A)** α-diversity of ND and D groups of swine fecal microbial communities in group A analyzed by a one-tailed Student’s *t*-test (*p*-value * < 0.05, ** < 0.01). Values are expressed as mean ± SEM. Ns: non-significance. **(B)** PCoA plots based on the weighted and unweighted UniFrac distances. Statistical analysis was performed using PERMANOVA. **(C)** Bar plots of selected genera that were significantly abundant in group A. Statistical analysis were performed using a one-tailed Student’s *t*-test (*p*-value * < 0.05).

To identify specific gut microbes related to the host physiological conditions, we compared the relative abundances of these genera between groups ND and D. There was no significant difference in the observed ASVs (*p* = 0.41) and phylogenetic diversity (*p* = 0.33) of microbial communities between the two groups; however, a higher Pielou’s evenness index was observed in group D (*p* = 0.01; Figure 3A). Furthermore, there was no statistically significant difference in both weighted (*p* = 0.65) and unweighted UniFrac (*p* = 0.38) distances between the two groups (Figure 3B). Among the increased genera (*Clostridium*, *Corynebacterium*, *Aerococcus*, and *Escherichia-Shigella*) in group A, only the abundance of *Corynebacterium* was significantly increased in group D (*p* = 0.04; Figure 3C). These results indicated that lincomycin administration increased the abundance of *Corynebacterium*, which may be closely related to swine diarrhea.

### Altered Gut Microbiota Follow Lincomycin Treatment Affects Host Health

**FIGURE 4:**
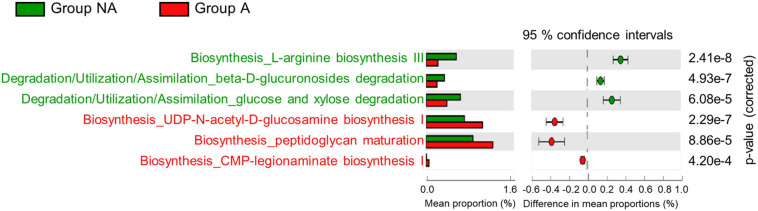
Predictive metagenomic analysis of functional profiling of swine fecal microbiota. Bacterial gene functions were predicted from the 16S rRNA gene-based microbial compositions using the PICRUSt2 algorithm and inferences from KEGG databases. Data from PICRUSt2 were imported into the STMAP package for statistical analysis and visualization. Only three pathways from each group were included in the *post hoc* plot.

It has been reported that antibiotic-induced alterations in gut microbiota influence host metabolism (Nieuwdorp et al., 2014). Hence, we performed PICRUSt2 analysis to predict the metabolic pathways in swine gut microbiota using 16S rRNA gene sequencing. As shown in Supplementary Figure 1, 57 KEGG pathways were estimated to have been affected by lincomycin administration (only those with *p*-values < 0.001 were included in the plot). The microbiome of group A exhibited enrichment in pathways related to the biosynthesis of peptidoglycan and lipopolysaccharide (UDP-*N*-acetyl-D-glucosamine biosynthesis I, peptidoglycan maturation, and CMP-legionaminate biosynthesis I). While it showed decrement on beta-D-glucuronides degradation, L-arginine biosynthesis III, and glucose and xylose degradation pathways (Figure 4), suggesting that the administration of lincomycin potentially affects the host metabolic pathways by changing the gut microbiota.

### Meta-Analysis of Swine Microbiome After Exposure to Diverse Antibiotics

**FIGURE 5:**
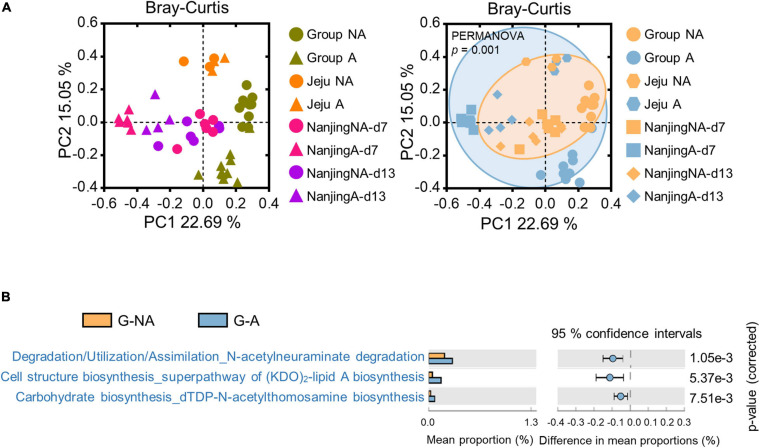
Meta-analysis of global swine gut microbiome datasets. The three datasets were downloaded using the SRA toolkit (one dataset was a study conducted in Jeju, Korea, and the other two were conducted in Nanjing, China). **(A)** β-diversity of global data based on the Bray-Curtis distance matrix. Statistical analysis was performed using PERMANOVA. The PCoA plot on the left is the result of displaying different colors for each study, and the result on the right is the result of displaying different colors depending on whether with or without antibiotics administration. **(B)** Bacterial gene functions were predicted using PICRUSt2 and imported into the STAMP package for statistical analysis and visualization. The *post hoc* plot showed the three putative pathways in group G-A. G-NA, Global non-administered group; G-A, Global administered group.

To further investigate the alteration of the gut microbiota in swine administered with diverse antibiotic types, including lincomycin, a meta-analysis was performed using publicly available data (chlortetracycline, sulfathiazole, and penicillin were used in SRP045387 and ampicillin, gentamycin, and metronidazole were used in SRP115601, Supplementary Table 2), which were derived from the feces of swine administered various types of antibiotics in geographically diverse regions. Merged-datasets were regrouped into G-NA (global non-administered) and G-A (global administered) groups (Supplementary Table 2). Bray-Curtis dissimilarities were ordinated and plotted by PCoA. The PERMANOVA test showed that the gut microbiota was significantly influenced by the administration of antibiotics (Figure 5A, *p* < 0.001). We then performed LEfSe using a logarithmic LDA score cut-off of 2.0, and identified 27 and 39 genera that were relatively more abundant in the microbiota of the G-NA and G-A groups, respectively. Some genera (e.g., *Treponema* and *Streptococcus*) were again identified to be more abundant in the G-NA group (Supplementary Figure 2). The PICRUSt2 analysis was performed to investigate the putative functional differences in the fecal microbiota between the G-NA and G-A groups. The top 20 most significantly different KEGG pathways in each group were included in the *post hoc* plot (Supplementary Figure 3). Overall, we detected enrichment of pathways involved in amino acid biosynthesis and nucleotide biosynthesis in group G-NA, and these functions were reduced in group G-A. In addition, there were significant differences in pathways related to virulence factor production, such as *N*-acetylneuraminate degradation, (KDO)_2_-lipid A biosynthesis, and dTDP-*N*-acetylthomosamine biosynthesis between G-NA and G-A (Figure 5B). Therefore, our results showed that the supplementation of the antibiotics used in our meta-analysis induced gut dysbiosis and changed the putative metabolic pathway.

## Discussion

Feeding sub-therapeutic doses of antimicrobials can adversely affect swine microbiota and health (Gresse et al., 2017; Zeineldin et al., 2019). The aim of this study was to identify the differences of the fecal microbiota and its predicted metabolic pathways between lincomycin-administered and non-administered finishing swine.

The loss microbial diversity or changes in relative abundance of the gut microbial community are referred to as gut dysbiosis (Wilkins et al., 2019). We found that lincomycin treatment induced the loss of microbial diversity (Figure 1B) and an alteration in the 4 major phyla (Figure 1D), such as Firmicutes, Bacteroidetes, Spirochetes, and Actinobacteria, which are dominant in swine gut (Becattini et al., 2016; Ke et al., 2019). The significant decrease in diversity and differences in the abundance of these bacteria suggest that swine gut microorganism environment may be disrupted by lincomycin administration. In addition, we observed a scattered distribution of microbial composition (Figure 1C), indicating that the administration of lincomycin might induce gut dysbiosis in finishing swine.

At the genus level, we detected lack of *Treponema*, *Succinivibrio*, *Fibrobacter*, and *Cellulosilyticum* abundance in the fecal microbiota of lincomycin-administered swine (Figure 2). These genera are widely known symbionts that play a role in the degradation of dietary fiber in the late stage of swine growth (Niu et al., 2015; Han et al., 2018; Makki et al., 2018; Tan et al., 2018). Fiber digestibility is an important function of the colon microbiota in finishing swine, because it releases energy and nutrients from indigestible materials (Lindberg, 2014; Jha et al., 2019). Previous studies have validated that the nutritional and health conditions, and fiber digestibility of swine are determined by the fiber properties of the feed, which correlated with the swine gut microbiota (Lindberg, 2014; Niu et al., 2015). Moreover, colonization resistance of host symbiotic gut microbes constitutes the first line of defense against invading pathogens, and this function depends on fiber digestibility of the commensal bacteria (Desai et al., 2016). Consistently, our results of the PICRUSt2 analysis revealed the decrease of pathways related to pectin (polysaccharide) degradation in lincomycin-administered swine (Figure 4). Furthermore, an enhancement of the L-arginine biosynthesis III pathway was observed in the microbiota of non-administered swine (Figure 4). Arginine, synthesized from glutamine, is required for growth performance and feed efficiency in growing swine (Hou and Wu, 2018) and enhances intestinal epithelial barrier function (Costa et al., 2014). These data suggest microbiota imbalance caused by lincomycin administration, which may affect the metabolic potential.

In the present study, an increased abundance of *Clostridium* and *Corynebacterium* was observed in lincomycin-administered swine fecal microbiota; this can be attributed to their antibiotic-resistant properties (Figure 2C). *Clostridium*, belonging to the phyla Firmicutes, contains around 100 species, including antibiotic-resistant bacteria and pathogenic species, such as *Clostridium perfringens* cause infectious disease in swine (Rood et al., 1985). Different *Corynebacterium* species have been isolated from sows with urinary tract infection and swine nasal swabs, which are resistant to multiple antibiotics (Vela et al., 2003; Poor et al., 2017). *Corynebacterium striatum* displays antibiotic resistance to many commonly used antimicrobials including clindamycin belonging to the class lincosamide (McMullen et al., 2017). Additionally, we observed a significant increase in the abundance of opportunistic pathogenic bacteria, including *Aerococcus* (Vela et al., 2007; Rasmussen, 2016) and *Escherichia-Shigella* (Zeineldin et al., 2019) in lincomycin-administered swine microbiota (Figure 2C). The propagation of these opportunistic pathogens and multidrug-resistant bacteria in swine gut microbiota suggests that lincomycin treatment may result in reduced colonization resistance, which can adversely affect swine health and growth. Here, the PICRUSt results of antibiotic-treated swine microbiota supported our hypothesis that sub-therapeutic dosages of antimicrobials reduce colonization resistance. An increase in metabolic pathways related to peptidoglycan maturation and CMP-legionaminate biosynthesis was observed in lincomycin-administered swine microbiota (Figure 4); these pathways are related to enhanced adherence of pathogenic bacteria to mammalian cell surfaces (Schoenhofen et al., 2009). However, a previous study that analyzed microbiota and antibiotic resistance genes in sows after feeding lincomycin, chlortetracycline, and amoxicillin for 12 days reported different results (Sun et al., 2014). These opposite results indicate that gut microbiota varies depending on the type of antibiotic, and an in-depth understanding of the alterations in swine gut microbiota caused by various antibiotics is necessary.

We therefore performed meta-analysis using other datasets to investigate the influence of multiple antimicrobials. The *post hoc* plot showed an increase in metabolic pathways related to biosynthesis of truncated lipopolysaccharides (Murphy et al., 2005), and synthesis of enterobacterial common antigen (Meier-Dieter et al., 1990) in antibiotic-administered swine (Figure 5B). Among these altered pathways, a notable metabolic alteration was observed in N-acetylneuraminate biosynthesis. N-acetylneuraminate, such as *N*-acetylglucosamine, N-mannosamine, and N-neuraminic acid, is an intermediate in the sialic acid degradation pathway, and sialidase activity is related to host intestinal mucin degradation, which is common in pathogenic bacteria (Vimr and Lichtensteiger, 2002). For example, *Salmonella* and *C. difficile* can utilize sialic acid from host mucus by utilizing microbiota-encoded sialidase enzymes during their enteric expansion (Ng et al., 2013). Although the antibiotics used in the references for meta-analysis have been commonly used to prevent swine colitis (Gao et al., 2018) or promote growth performance (Unno et al., 2015), the remarkable increase in specific gut microbes and metabolic pathways associated with pathogenicity maintenance and host intestinal invasion of pathogens in antibiotic-administered swine suggest that compatibility of antimicrobial supplements in the swine industry.

In conclusion, we demonstrated that in-feed subtherapeutic doses of lincomycin disrupted the structure and metabolic function of the finishing swine gut microbiome, which may adversely affect swine health. This study suggests that antibiotic administration potentially influences host colonization resistance and nutrient digestion in the swine microbiota. Our findings reveal the effect of lincomycin on finishing swine gut microbiota and suggest that the development of antibiotic alternatives is needed to improve swine growth and health. Further study with a large number of samples would be necessary to fully understand the relationship between lincomycin-induced dysbiosis and swine health.

## Data Availability Statement

The datasets generated for this study can be found in the NCBI GenBank, and its accession number was PRJNA643361.

## Ethics Statement

The animal study was reviewed and approved by all experimental procedures were conducted by the guidelines approved by the Institutional Animal Care and Use Committee (IACUC) at the National Institute of Animal Science (approved no. NIAS-2019119). Written informed consent was obtained from the owners for the participation of their animals in this study.

## Author Contributions

S-HK, H-JC, and HEJ conceived and designed the experiments. HEJ, M-SK, TWW, DWK, and JL performed the experiments. HEJ, M-SK, TWW, MY, JL, M-YS, S-HK, and H-JC analyzed the data. HEJ, S-HK, and H-JC prepared the manuscript. S-HK and H-JC supervised the study. All authors contributed to the article and approved the submitted version.

## Conflict of Interest

The authors declare that the research was conducted in the absence of any commercial or financial relationships that could be construed as a potential conflict of interest.

## Footnotes

1 https://docs.qiime2.org/2019.4/

2 https://github.com/qiime2/q2-diversity

3 https://github.com/qiime2/q2-featureclassifier

4 https://github.com/qiime2/q2-taxa

5 https://www.ncbi.nlm.nih.gov/sra
