# Supplementary material for: Alteration of Gut Microbiota After Antibiotic Exposure in Finishing Swine
Source: Front Microbiol. 2021 Feb 12;12:596002. doi: 10.3389/fmicb.2021.596002 (PMC7906994; doi:10.3389/fmicb.2021.596002)
Supplement: Supplementary file 1 [file Presentation_1.pptx]

## Slide 1
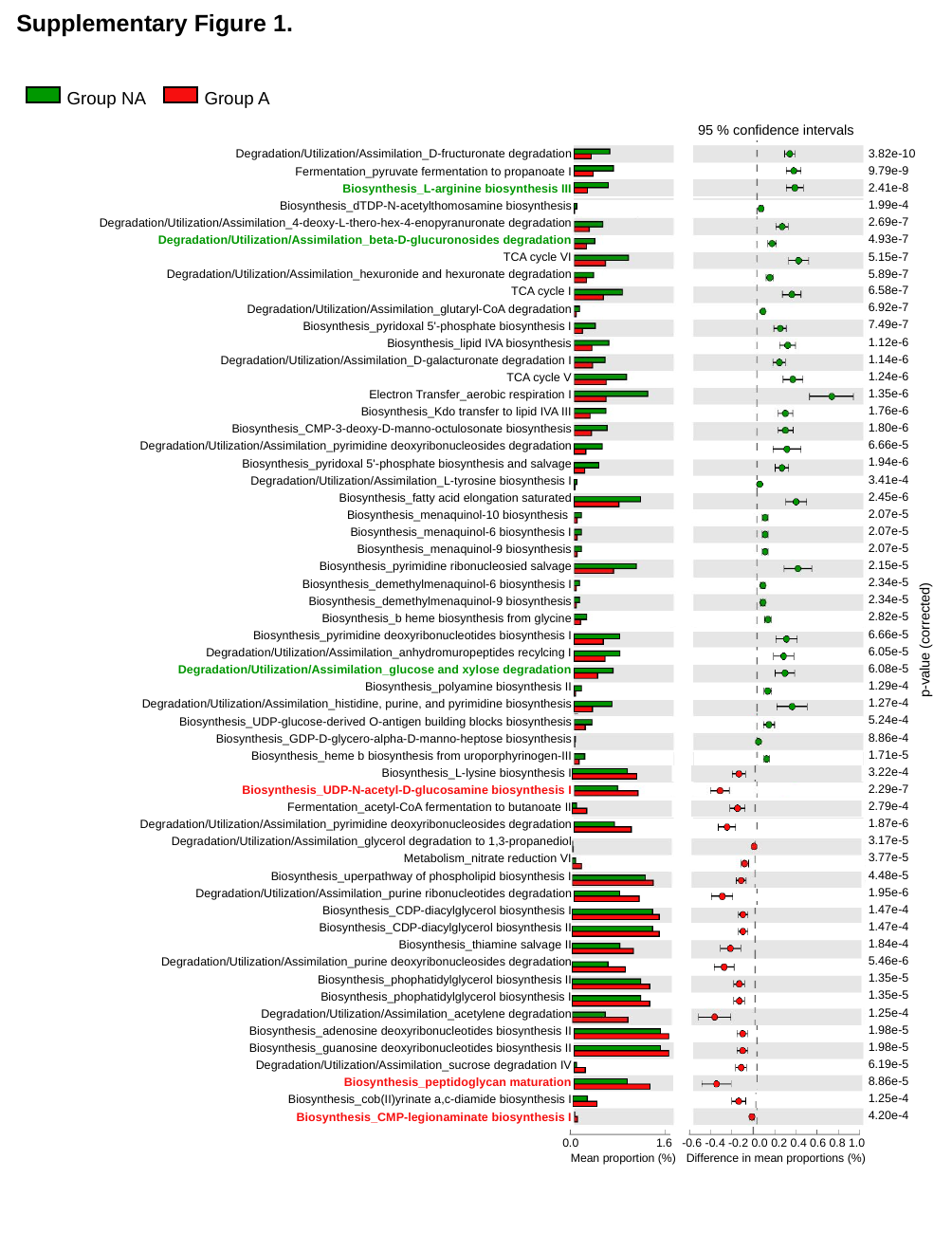

Supplementary Figure 1.
Group NA
Group A
95 % confidence intervals
| 3.82e-10 |
| --- |
| 9.79e-9 |
| 2.41e-8 |
| 1.99e-4 |
| 2.69e-7 |
| 4.93e-7 |
| 5.15e-7 |
| 5.89e-7 |
| 6.58e-7 |
| 6.92e-7 |
| 7.49e-7 |
| 1.12e-6 |
| 1.14e-6 |
| 1.24e-6 |
| 1.35e-6 |
| 1.76e-6 |
| 1.80e-6 |
| 6.66e-5 |
| 1.94e-6 |
| 3.41e-4 |
| 2.45e-6 |
| 2.07e-5 |
| 2.07e-5 |
| 2.07e-5 |
| 2.15e-5 |
| 2.34e-5 |
| 2.34e-5 |
| 2.82e-5 |
| 6.66e-5 |
| 6.05e-5 |
| 6.08e-5 |
| 1.29e-4 |
| 1.27e-4 |
| 5.24e-4 |
| 8.86e-4 |
| 1.71e-5 |
| 3.22e-4 |
| 2.29e-7 |
| 2.79e-4 |
| 1.87e-6 |
| 3.17e-5 |
| 3.77e-5 |
| 4.48e-5 |
| 1.95e-6 |
| 1.47e-4 |
| 1.47e-4 |
| 1.84e-4 |
| 5.46e-6 |
| 1.35e-5 |
| 1.35e-5 |
| 1.25e-4 |
| 1.98e-5 |
| 1.98e-5 |
| 6.19e-5 |
| 8.86e-5 |
| 1.25e-4 |
| 4.20e-4 |
| Degradation/Utilization/Assimilation\_D-fructuronate degradation |
| --- |
| Fermentation\_pyruvate fermentation to propanoate I |
| Biosynthesis\_L-arginine biosynthesis III |
| Biosynthesis\_dTDP-N-acetylthomosamine biosynthesis |
| Degradation/Utilization/Assimilation\_4-deoxy-L-thero-hex-4-enopyranuronate degradation |
| Degradation/Utilization/Assimilation\_beta-D-glucuronosides degradation |
| TCA cycle VI |
| Degradation/Utilization/Assimilation\_hexuronide and hexuronate degradation |
| TCA cycle I |
| Degradation/Utilization/Assimilation\_glutaryl-CoA degradation |
| Biosynthesis\_pyridoxal 5'-phosphate biosynthesis I |
| Biosynthesis\_lipid IVA biosynthesis |
| Degradation/Utilization/Assimilation\_D-galacturonate degradation I |
| TCA cycle V |
| Electron Transfer\_aerobic respiration I |
| Biosynthesis\_Kdo transfer to lipid IVA III |
| Biosynthesis\_CMP-3-deoxy-D-manno-octulosonate biosynthesis |
| Degradation/Utilization/Assimilation\_pyrimidine deoxyribonucleosides degradation |
| Biosynthesis\_pyridoxal 5'-phosphate biosynthesis and salvage |
| Degradation/Utilization/Assimilation\_L-tyrosine biosynthesis I |
| Biosynthesis\_fatty acid elongation saturated |
| Biosynthesis\_menaquinol-10 biosynthesis |
| Biosynthesis\_menaquinol-6 biosynthesis I |
| Biosynthesis\_menaquinol-9 biosynthesis |
| Biosynthesis\_pyrimidine ribonucleosied salvage |
| Biosynthesis\_demethylmenaquinol-6 biosynthesis I |
| Biosynthesis\_demethylmenaquinol-9 biosynthesis |
| Biosynthesis\_b heme biosynthesis from glycine |
| Biosynthesis\_pyrimidine deoxyribonucleotides biosynthesis I |
| Degradation/Utilization/Assimilation\_anhydromuropeptides recylcing I |
| Degradation/Utilization/Assimilation\_glucose and xylose degradation |
| Biosynthesis\_polyamine biosynthesis II |
| Degradation/Utilization/Assimilation\_histidine, purine, and pyrimidine biosynthesis |
| Biosynthesis\_UDP-glucose-derived O-antigen building blocks biosynthesis |
| Biosynthesis\_GDP-D-glycero-alpha-D-manno-heptose biosynthesis |
| Biosynthesis\_heme b biosynthesis from uroporphyrinogen-III |
| Biosynthesis\_L-lysine biosynthesis I |
| Biosynthesis\_UDP-N-acetyl-D-glucosamine biosynthesis I |
| Fermentation\_acetyl-CoA fermentation to butanoate II |
| Degradation/Utilization/Assimilation\_pyrimidine deoxyribonucleosides degradation |
| Degradation/Utilization/Assimilation\_glycerol degradation to 1,3-propanediol |
| Metabolism\_nitrate reduction VI |
| Biosynthesis\_uperpathway of phospholipid biosynthesis I |
| Degradation/Utilization/Assimilation\_purine ribonucleotides degradation |
| Biosynthesis\_CDP-diacylglycerol biosynthesis I |
| Biosynthesis\_CDP-diacylglycerol biosynthesis II |
| Biosynthesis\_thiamine salvage II |
| Degradation/Utilization/Assimilation\_purine deoxyribonucleosides degradation |
| Biosynthesis\_phophatidylglycerol biosynthesis II |
| Biosynthesis\_phophatidylglycerol biosynthesis I |
| Degradation/Utilization/Assimilation\_acetylene degradation |
| Biosynthesis\_adenosine deoxyribonucleotides biosynthesis II |
| Biosynthesis\_guanosine deoxyribonucleotides biosynthesis II |
| Degradation/Utilization/Assimilation\_sucrose degradation IV |
| Biosynthesis\_peptidoglycan maturation |
| Biosynthesis\_cob(II)yrinate a,c-diamide biosynthesis I |
| Biosynthesis\_CMP-legionaminate biosynthesis I |
p-value (corrected)
0.0
1.6
-0.6 -0.4 -0.2 0.0
0.2 0.4 0.6 0.8 1.0
Mean proportion (%)
Difference in mean proportions (%)

## Slide 2
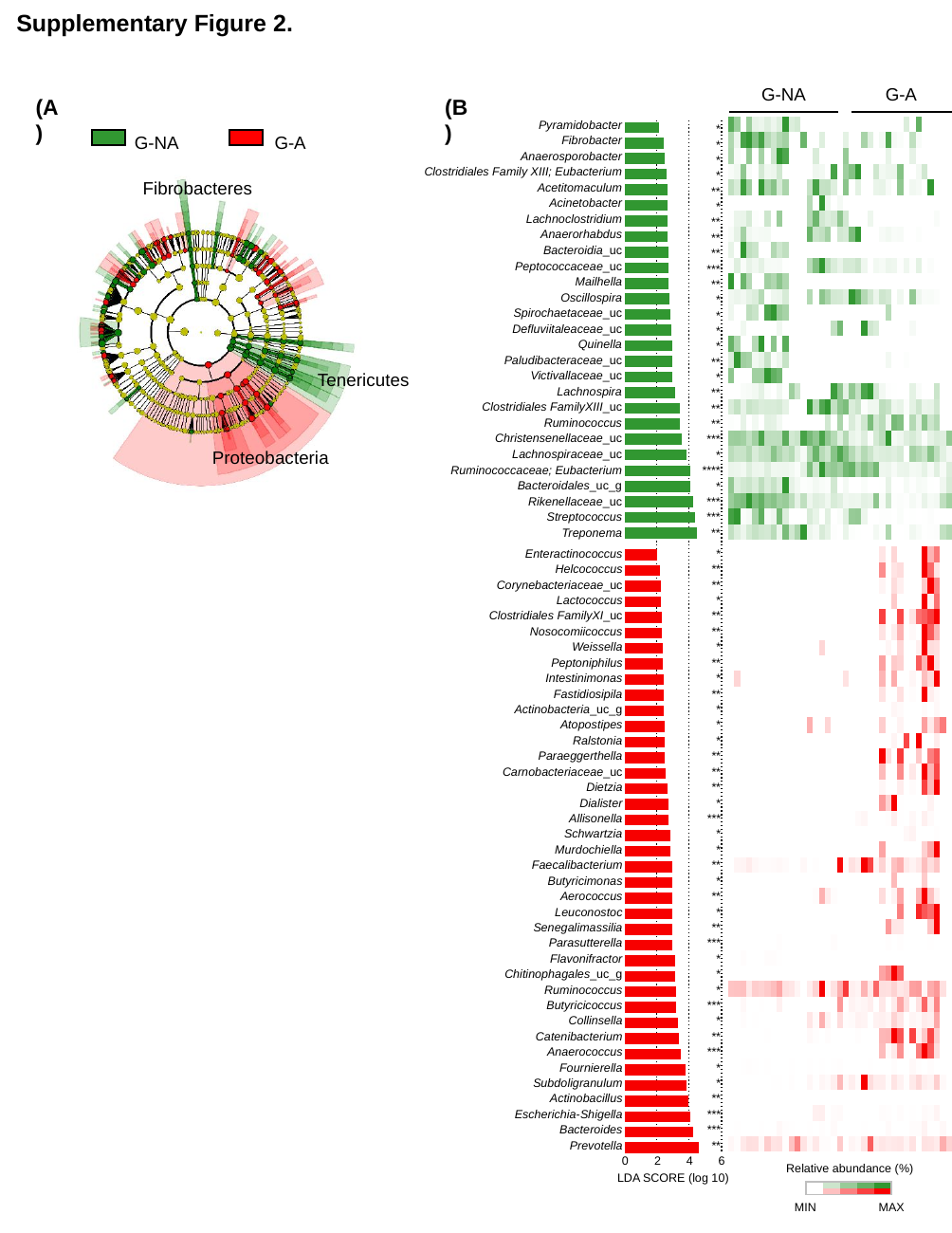

Supplementary Figure 2.
G-NA
G-A
(A)
(B)
| | | | | | | | | | | | | | | | | | | | | | | | | | | | | | | | | | | | | | | | | | | | | | | | |
| --- | --- | --- | --- | --- | --- | --- | --- | --- | --- | --- | --- | --- | --- | --- | --- | --- | --- | --- | --- | --- | --- | --- | --- | --- | --- | --- | --- | --- | --- | --- | --- | --- | --- | --- | --- | --- | --- | --- | --- | --- | --- | --- | --- | --- | --- | --- | --- |
| | | | | | | | | | | | | | | | | | | | | | | | | | | | | | | | | | | | | | | | | | | | | | | | |
| | | | | | | | | | | | | | | | | | | | | | | | | | | | | | | | | | | | | | | | | | | | | | | | |
| | | | | | | | | | | | | | | | | | | | | | | | | | | | | | | | | | | | | | | | | | | | | | | | |
| | | | | | | | | | | | | | | | | | | | | | | | | | | | | | | | | | | | | | | | | | | | | | | | |
| | | | | | | | | | | | | | | | | | | | | | | | | | | | | | | | | | | | | | | | | | | | | | | | |
| | | | | | | | | | | | | | | | | | | | | | | | | | | | | | | | | | | | | | | | | | | | | | | | |
| | | | | | | | | | | | | | | | | | | | | | | | | | | | | | | | | | | | | | | | | | | | | | | | |
| | | | | | | | | | | | | | | | | | | | | | | | | | | | | | | | | | | | | | | | | | | | | | | | |
| | | | | | | | | | | | | | | | | | | | | | | | | | | | | | | | | | | | | | | | | | | | | | | | |
| | | | | | | | | | | | | | | | | | | | | | | | | | | | | | | | | | | | | | | | | | | | | | | | |
| | | | | | | | | | | | | | | | | | | | | | | | | | | | | | | | | | | | | | | | | | | | | | | | |
| | | | | | | | | | | | | | | | | | | | | | | | | | | | | | | | | | | | | | | | | | | | | | | | |
| | | | | | | | | | | | | | | | | | | | | | | | | | | | | | | | | | | | | | | | | | | | | | | | |
| | | | | | | | | | | | | | | | | | | | | | | | | | | | | | | | | | | | | | | | | | | | | | | | |
| | | | | | | | | | | | | | | | | | | | | | | | | | | | | | | | | | | | | | | | | | | | | | | | |
| | | | | | | | | | | | | | | | | | | | | | | | | | | | | | | | | | | | | | | | | | | | | | | | |
| | | | | | | | | | | | | | | | | | | | | | | | | | | | | | | | | | | | | | | | | | | | | | | | |
| | | | | | | | | | | | | | | | | | | | | | | | | | | | | | | | | | | | | | | | | | | | | | | | |
| | | | | | | | | | | | | | | | | | | | | | | | | | | | | | | | | | | | | | | | | | | | | | | | |
| | | | | | | | | | | | | | | | | | | | | | | | | | | | | | | | | | | | | | | | | | | | | | | | |
| | | | | | | | | | | | | | | | | | | | | | | | | | | | | | | | | | | | | | | | | | | | | | | | |
| | | | | | | | | | | | | | | | | | | | | | | | | | | | | | | | | | | | | | | | | | | | | | | | |
| | | | | | | | | | | | | | | | | | | | | | | | | | | | | | | | | | | | | | | | | | | | | | | | |
| | | | | | | | | | | | | | | | | | | | | | | | | | | | | | | | | | | | | | | | | | | | | | | | |
| | | | | | | | | | | | | | | | | | | | | | | | | | | | | | | | | | | | | | | | | | | | | | | | |
| | | | | | | | | | | | | | | | | | | | | | | | | | | | | | | | | | | | | | | | | | | | | | | | |
| Pyramidobacter |
| --- |
| Fibrobacter |
| Anaerosporobacter |
| Clostridiales Family XIII; Eubacterium |
| Acetitomaculum |
| Acinetobacter |
| Lachnoclostridium |
| Anaerorhabdus |
| Bacteroidia\_uc |
| Peptococcaceae\_uc |
| Mailhella |
| Oscillospira |
| Spirochaetaceae\_uc |
| Defluviitaleaceae\_uc |
| Quinella |
| Paludibacteraceae\_uc |
| Victivallaceae\_uc |
| Lachnospira |
| Clostridiales FamilyXIII\_uc |
| Ruminococcus |
| Christensenellaceae\_uc |
| Lachnospiraceae\_uc |
| Ruminococcaceae; Eubacterium |
| Bacteroidales\_uc\_g |
| Rikenellaceae\_uc |
| Streptococcus |
| Treponema |
G-NA
G-A
| \* |
| --- |
| \* |
| \* |
| \* |
| \*\* |
| \* |
| \*\* |
| \*\* |
| \*\* |
| \*\*\* |
| \*\* |
| \* |
| \* |
| \* |
| \* |
| \*\* |
| \* |
| \*\* |
| \*\* |
| \*\* |
| \*\*\* |
| \* |
| \*\*\*\* |
| \* |
| \*\*\* |
| \*\*\* |
| \*\* |
Fibrobacteres
Tenericutes
Proteobacteria
| Enteractinococcus |
| --- |
| Helcococcus |
| Corynebacteriaceae\_uc |
| Lactococcus |
| Clostridiales FamilyXI\_uc |
| Nosocomiicoccus |
| Weissella |
| Peptoniphilus |
| Intestinimonas |
| Fastidiosipila |
| Actinobacteria\_uc\_g |
| Atopostipes |
| Ralstonia |
| Paraeggerthella |
| Carnobacteriaceae\_uc |
| Dietzia |
| Dialister |
| Allisonella |
| Schwartzia |
| Murdochiella |
| Faecalibacterium |
| Butyricimonas |
| Aerococcus |
| Leuconostoc |
| Senegalimassilia |
| Parasutterella |
| Flavonifractor |
| Chitinophagales\_uc\_g |
| Ruminococcus |
| Butyricicoccus |
| Collinsella |
| Catenibacterium |
| Anaerococcus |
| Fournierella |
| Subdoligranulum |
| Actinobacillus |
| Escherichia-Shigella |
| Bacteroides |
| Prevotella |
| \* |
| --- |
| \*\* |
| \*\* |
| \* |
| \*\* |
| \*\* |
| \* |
| \*\* |
| \* |
| \*\* |
| \* |
| \* |
| \* |
| \*\* |
| \*\* |
| \*\* |
| \* |
| \*\*\* |
| \* |
| \* |
| \*\* |
| \* |
| \*\* |
| \* |
| \*\* |
| \*\*\* |
| \* |
| \* |
| \* |
| \*\*\* |
| \* |
| \*\* |
| \*\*\* |
| \* |
| \* |
| \*\* |
| \*\*\* |
| \*\*\* |
| \*\* |
| | | | | | | | | | | | | | | | | | | | | | | | | | | | | | | | | | | | | | | | | | | | | | | | |
| --- | --- | --- | --- | --- | --- | --- | --- | --- | --- | --- | --- | --- | --- | --- | --- | --- | --- | --- | --- | --- | --- | --- | --- | --- | --- | --- | --- | --- | --- | --- | --- | --- | --- | --- | --- | --- | --- | --- | --- | --- | --- | --- | --- | --- | --- | --- | --- |
| | | | | | | | | | | | | | | | | | | | | | | | | | | | | | | | | | | | | | | | | | | | | | | | |
| | | | | | | | | | | | | | | | | | | | | | | | | | | | | | | | | | | | | | | | | | | | | | | | |
| | | | | | | | | | | | | | | | | | | | | | | | | | | | | | | | | | | | | | | | | | | | | | | | |
| | | | | | | | | | | | | | | | | | | | | | | | | | | | | | | | | | | | | | | | | | | | | | | | |
| | | | | | | | | | | | | | | | | | | | | | | | | | | | | | | | | | | | | | | | | | | | | | | | |
| | | | | | | | | | | | | | | | | | | | | | | | | | | | | | | | | | | | | | | | | | | | | | | | |
| | | | | | | | | | | | | | | | | | | | | | | | | | | | | | | | | | | | | | | | | | | | | | | | |
| | | | | | | | | | | | | | | | | | | | | | | | | | | | | | | | | | | | | | | | | | | | | | | | |
| | | | | | | | | | | | | | | | | | | | | | | | | | | | | | | | | | | | | | | | | | | | | | | | |
| | | | | | | | | | | | | | | | | | | | | | | | | | | | | | | | | | | | | | | | | | | | | | | | |
| | | | | | | | | | | | | | | | | | | | | | | | | | | | | | | | | | | | | | | | | | | | | | | | |
| | | | | | | | | | | | | | | | | | | | | | | | | | | | | | | | | | | | | | | | | | | | | | | | |
| | | | | | | | | | | | | | | | | | | | | | | | | | | | | | | | | | | | | | | | | | | | | | | | |
| | | | | | | | | | | | | | | | | | | | | | | | | | | | | | | | | | | | | | | | | | | | | | | | |
| | | | | | | | | | | | | | | | | | | | | | | | | | | | | | | | | | | | | | | | | | | | | | | | |
| | | | | | | | | | | | | | | | | | | | | | | | | | | | | | | | | | | | | | | | | | | | | | | | |
| | | | | | | | | | | | | | | | | | | | | | | | | | | | | | | | | | | | | | | | | | | | | | | | |
| | | | | | | | | | | | | | | | | | | | | | | | | | | | | | | | | | | | | | | | | | | | | | | | |
| | | | | | | | | | | | | | | | | | | | | | | | | | | | | | | | | | | | | | | | | | | | | | | | |
| | | | | | | | | | | | | | | | | | | | | | | | | | | | | | | | | | | | | | | | | | | | | | | | |
| | | | | | | | | | | | | | | | | | | | | | | | | | | | | | | | | | | | | | | | | | | | | | | | |
| | | | | | | | | | | | | | | | | | | | | | | | | | | | | | | | | | | | | | | | | | | | | | | | |
| | | | | | | | | | | | | | | | | | | | | | | | | | | | | | | | | | | | | | | | | | | | | | | | |
| | | | | | | | | | | | | | | | | | | | | | | | | | | | | | | | | | | | | | | | | | | | | | | | |
| | | | | | | | | | | | | | | | | | | | | | | | | | | | | | | | | | | | | | | | | | | | | | | | |
| | | | | | | | | | | | | | | | | | | | | | | | | | | | | | | | | | | | | | | | | | | | | | | | |
| | | | | | | | | | | | | | | | | | | | | | | | | | | | | | | | | | | | | | | | | | | | | | | | |
| | | | | | | | | | | | | | | | | | | | | | | | | | | | | | | | | | | | | | | | | | | | | | | | |
| | | | | | | | | | | | | | | | | | | | | | | | | | | | | | | | | | | | | | | | | | | | | | | | |
| | | | | | | | | | | | | | | | | | | | | | | | | | | | | | | | | | | | | | | | | | | | | | | | |
| | | | | | | | | | | | | | | | | | | | | | | | | | | | | | | | | | | | | | | | | | | | | | | | |
| | | | | | | | | | | | | | | | | | | | | | | | | | | | | | | | | | | | | | | | | | | | | | | | |
| | | | | | | | | | | | | | | | | | | | | | | | | | | | | | | | | | | | | | | | | | | | | | | | |
| | | | | | | | | | | | | | | | | | | | | | | | | | | | | | | | | | | | | | | | | | | | | | | | |
| | | | | | | | | | | | | | | | | | | | | | | | | | | | | | | | | | | | | | | | | | | | | | | | |
| | | | | | | | | | | | | | | | | | | | | | | | | | | | | | | | | | | | | | | | | | | | | | | | |
| | | | | | | | | | | | | | | | | | | | | | | | | | | | | | | | | | | | | | | | | | | | | | | | |
| | | | | | | | | | | | | | | | | | | | | | | | | | | | | | | | | | | | | | | | | | | | | | | | |
0
2
4
6
Relative abundance (%)
LDA SCORE (log 10)
| | | | | |
| --- | --- | --- | --- | --- |
| | | | | |
MIN
MAX

## Slide 3
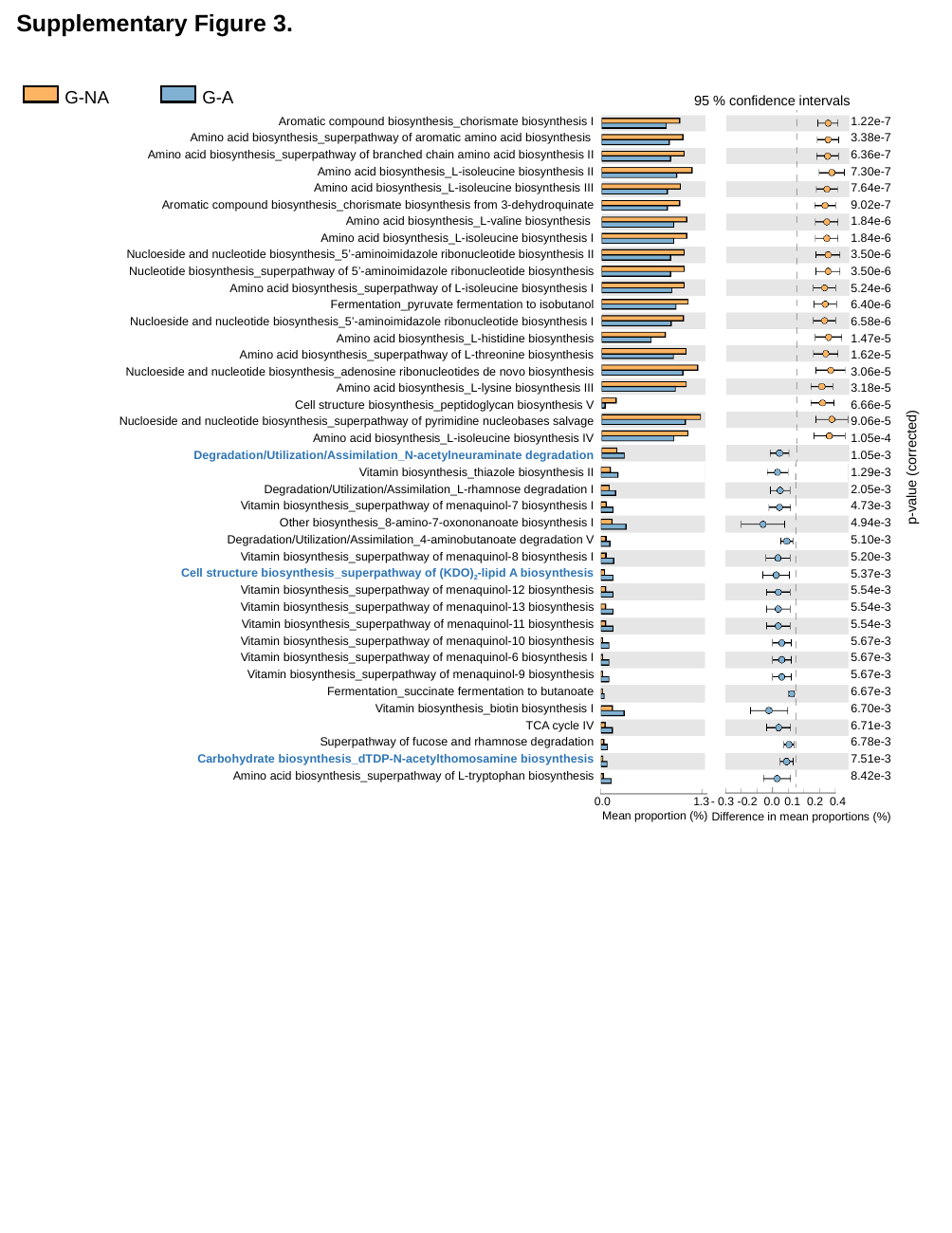

Supplementary Figure 3.
G-NA
G-A
95 % confidence intervals
| 1.22e-7 |
| --- |
| 3.38e-7 |
| 6.36e-7 |
| 7.30e-7 |
| 7.64e-7 |
| 9.02e-7 |
| 1.84e-6 |
| 1.84e-6 |
| 3.50e-6 |
| 3.50e-6 |
| 5.24e-6 |
| 6.40e-6 |
| 6.58e-6 |
| 1.47e-5 |
| 1.62e-5 |
| 3.06e-5 |
| 3.18e-5 |
| 6.66e-5 |
| 9.06e-5 |
| 1.05e-4 |
| Aromatic compound biosynthesis\_chorismate biosynthesis I |
| --- |
| Amino acid biosynthesis\_superpathway of aromatic amino acid biosynthesis |
| Amino acid biosynthesis\_superpathway of branched chain amino acid biosynthesis II |
| Amino acid biosynthesis\_L-isoleucine biosynthesis II |
| Amino acid biosynthesis\_L-isoleucine biosynthesis III |
| Aromatic compound biosynthesis\_chorismate biosynthesis from 3-dehydroquinate |
| Amino acid biosynthesis\_L-valine biosynthesis |
| Amino acid biosynthesis\_L-isoleucine biosynthesis I |
| Nucloeside and nucleotide biosynthesis\_5’-aminoimidazole ribonucleotide biosynthesis II |
| Nucleotide biosynthesis\_superpathway of 5’-aminoimidazole ribonucleotide biosynthesis |
| Amino acid biosynthesis\_superpathway of L-isoleucine biosynthesis I |
| Fermentation\_pyruvate fermentation to isobutanol |
| Nucloeside and nucleotide biosynthesis\_5’-aminoimidazole ribonucleotide biosynthesis I |
| Amino acid biosynthesis\_L-histidine biosynthesis |
| Amino acid biosynthesis\_superpathway of L-threonine biosynthesis |
| Nucloeside and nucleotide biosynthesis\_adenosine ribonucleotides de novo biosynthesis |
| Amino acid biosynthesis\_L-lysine biosynthesis III |
| Cell structure biosynthesis\_peptidoglycan biosynthesis V |
| Nucloeside and nucleotide biosynthesis\_superpathway of pyrimidine nucleobases salvage |
| Amino acid biosynthesis\_L-isoleucine biosynthesis IV |
| 1.05e-3 |
| --- |
| 1.29e-3 |
| 2.05e-3 |
| 4.73e-3 |
| 4.94e-3 |
| 5.10e-3 |
| 5.20e-3 |
| 5.37e-3 |
| 5.54e-3 |
| 5.54e-3 |
| 5.54e-3 |
| 5.67e-3 |
| 5.67e-3 |
| 5.67e-3 |
| 6.67e-3 |
| 6.70e-3 |
| 6.71e-3 |
| 6.78e-3 |
| 7.51e-3 |
| 8.42e-3 |
| Degradation/Utilization/Assimilation\_N-acetylneuraminate degradation |
| --- |
| Vitamin biosynthesis\_thiazole biosynthesis II |
| Degradation/Utilization/Assimilation\_L-rhamnose degradation I |
| Vitamin biosynthesis\_superpathway of menaquinol-7 biosynthesis I |
| Other biosynthesis\_8-amino-7-oxononanoate biosynthesis I |
| Degradation/Utilization/Assimilation\_4-aminobutanoate degradation V |
| Vitamin biosynthesis\_superpathway of menaquinol-8 biosynthesis I |
| Cell structure biosynthesis\_superpathway of (KDO)2-lipid A biosynthesis |
| Vitamin biosynthesis\_superpathway of menaquinol-12 biosynthesis |
| Vitamin biosynthesis\_superpathway of menaquinol-13 biosynthesis |
| Vitamin biosynthesis\_superpathway of menaquinol-11 biosynthesis |
| Vitamin biosynthesis\_superpathway of menaquinol-10 biosynthesis |
| Vitamin biosynthesis\_superpathway of menaquinol-6 biosynthesis I |
| Vitamin biosynthesis\_superpathway of menaquinol-9 biosynthesis |
| Fermentation\_succinate fermentation to butanoate |
| Vitamin biosynthesis\_biotin biosynthesis I |
| TCA cycle IV |
| Superpathway of fucose and rhamnose degradation |
| Carbohydrate biosynthesis\_dTDP-N-acetylthomosamine biosynthesis |
| Amino acid biosynthesis\_superpathway of L-tryptophan biosynthesis |
p-value (corrected)
- 0.3 -0.2 0.0
0.0
1.3
0.1 0.2 0.4
Mean proportion (%)
Difference in mean proportions (%)

## Slide 4
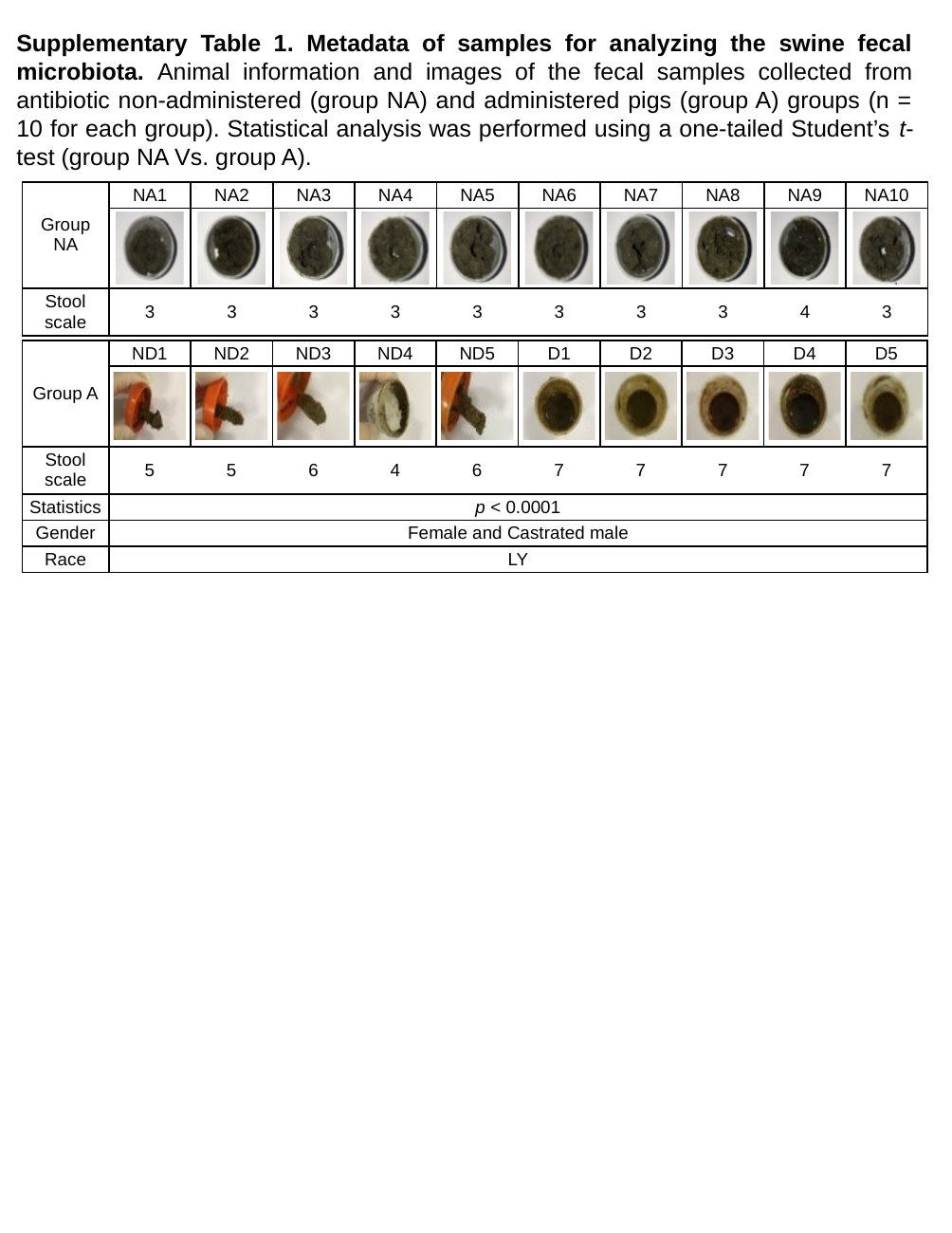

Supplementary Table 1. Metadata of samples for analyzing the swine fecal microbiota. Animal information and images of the fecal samples collected from antibiotic non-administered (group NA) and administered pigs (group A) groups (n = 10 for each group). Statistical analysis was performed using a one-tailed Student’s t-test (group NA Vs. group A).
| Group NA | NA1 | NA2 | NA3 | NA4 | NA5 | NA6 | NA7 | NA8 | NA9 | NA10 |
| --- | --- | --- | --- | --- | --- | --- | --- | --- | --- | --- |
| | | | | | | | | | | |
| Stool scale | 3 | 3 | 3 | 3 | 3 | 3 | 3 | 3 | 4 | 3 |
| Group A | ND1 | ND2 | ND3 | ND4 | ND5 | D1 | D2 | D3 | D4 | D5 |
| --- | --- | --- | --- | --- | --- | --- | --- | --- | --- | --- |
| | | | | | | | | | | |
| Stool scale | 5 | 5 | 6 | 4 | 6 | 7 | 7 | 7 | 7 | 7 |
| Statistics | p < 0.0001 | | | | | | | | | |
| Gender | Female and Castrated male | | | | | | | | | |
| Race | LY | | | | | | | | | |

## Slide 5
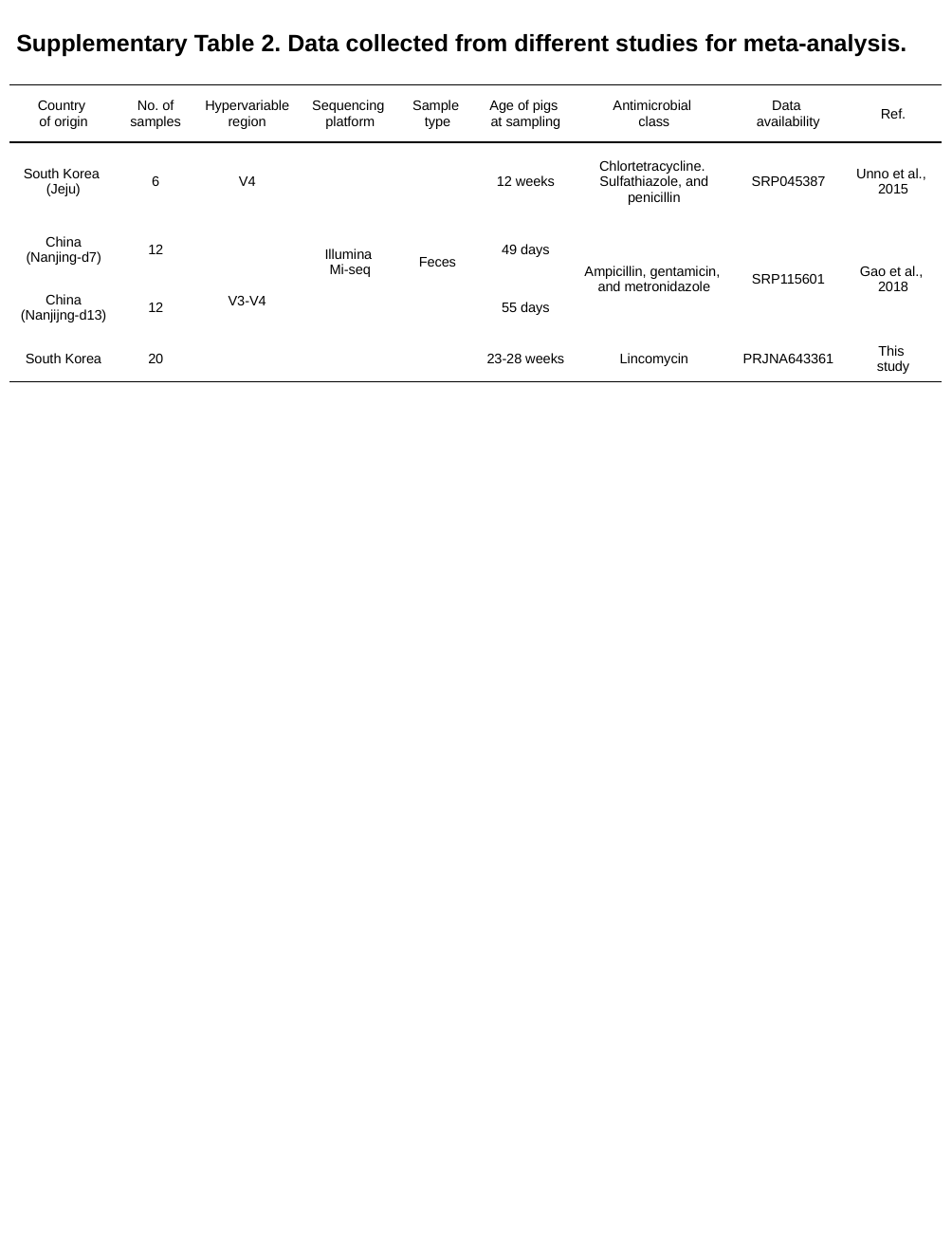

Supplementary Table 2. Data collected from different studies for meta-analysis.
| Country of origin | No. of samples | Hypervariable region | Sequencing platform | Sample type | Age of pigs at sampling | Antimicrobial class | Data availability | Ref. |
| --- | --- | --- | --- | --- | --- | --- | --- | --- |
| South Korea (Jeju) | 6 | V4 | Illumina Mi-seq | Feces | 12 weeks | Chlortetracycline. Sulfathiazole, and penicillin | SRP045387 | Unno et al., 2015 |
| China (Nanjing-d7) | 12 | V3-V4 | | | 49 days | Ampicillin, gentamicin, and metronidazole | SRP115601 | Gao et al., 2018 |
| China (Nanjijng-d13) | 12 | | | | 55 days | | | |
| South Korea | 20 | | | | 23-28 weeks | Lincomycin | PRJNA643361 | This study |

## Slide 6
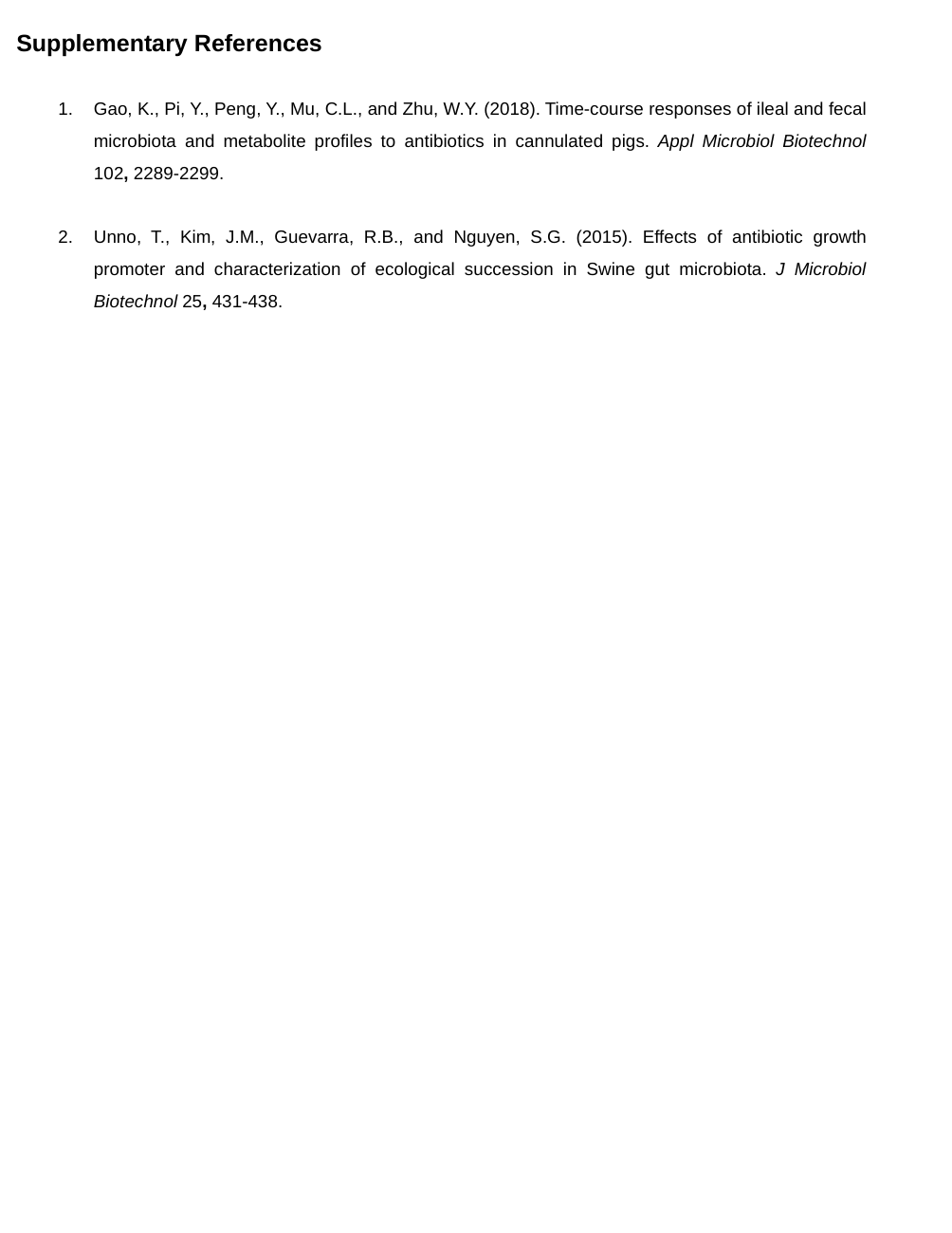

Supplementary References
Gao, K., Pi, Y., Peng, Y., Mu, C.L., and Zhu, W.Y. (2018). Time-course responses of ileal and fecal microbiota and metabolite profiles to antibiotics in cannulated pigs. Appl Microbiol Biotechnol 102, 2289-2299.
Unno, T., Kim, J.M., Guevarra, R.B., and Nguyen, S.G. (2015). Effects of antibiotic growth promoter and characterization of ecological succession in Swine gut microbiota. J Microbiol Biotechnol 25, 431-438.
